# Evidence-based design recommendations for prevalence studies on multimorbidity: improving comparability of estimates

**DOI:** 10.1186/s12963-017-0126-4

**Published:** 2017-03-07

**Authors:** Barbara M. Holzer, Klarissa Siebenhuener, Matthias Bopp, Christoph E. Minder

**Affiliations:** 1 Department of Internal Medicine, University Hospital Zurich, Zurich, Switzerland; 2 Epidemiology, Biostatistics and Prevention Institute, University of Zurich, Zurich, Switzerland; 3 Horten Center, University Hospital Zurich, Zurich, Switzerland; 4 Center of Competence Multimorbidity, University of Zurich, Zurich, Switzerland; 5 University Research Priority Program ‘Dynamics of Healthy Aging’, University of Zurich, Zurich, Switzerland

**Keywords:** Age, Gender, Study design variables, Multiple chronic conditions, Systematic review

## Abstract

**Background:**

In aging populations, multimorbidity causes a disease burden of growing importance and cost. However, estimates of the prevalence of multimorbidity (prevMM) vary widely across studies, impeding valid comparisons and interpretation of differences. With this study we pursued two research objectives: (1) to identify a set of study design and demographic factors related to prevMM, and (2) based on (1), to formulate design recommendations for future studies with improved comparability of prevalence estimates.

**Methods:**

Study data were obtained through systematic review of the literature. UsingPubMed/MEDLINE, Embase, CINAHL, Web of Science, BIOSIS, and Google Scholar, we looked for articles with the terms “multimorbidity,” “comorbidity,” “polymorbidity,” and variations of these published in English or German in the years 1990 to 2011. We selected quantitative studies of the prevalence of multimorbidity (two or more chronic medical conditions) with a minimum sample size of 50 and a study population with a majority of Caucasians. Our database consisted of prevalence estimates in 108 age groups taken from 45 studies. To assess the effects of study design variables, we used meta regression models.

**Results:**

In 58% of the studies, there was only one age group, i.e., no stratification by age. The number of persons per age group ranged from 136 to 5.6 million. Our analyses identified the following variables as highly significant: “mean age,” “number of age groups”, and “data reporting quality” (all *p* < 0.0001). “Setting,” “disease classification,” and “number of diseases in the classification” were significant (0.01 < *p* ≤ 0.03), and “data collection period” and “data source” were non-significant. A separate analysis showed that prevMM was significantly higher in women than men (sign test, *p* = 0.0015).

**Conclusions:**

Comparable prevalence estimates are urgently needed for realistic description of the magnitude of the problem of multimorbidity. Based on the results of our analyses of variables affecting prevMM, we make some design recommendations. Our suggestions were guided by a pragmatic approach and aimed at facilitating the implementation of a uniform methodology. This should aid progress towards a more uniform operationalization of multimorbidity.

**Electronic supplementary material:**

The online version of this article (doi:10.1186/s12963-017-0126-4) contains supplementary material, which is available to authorized users.

## Background

Multimorbidity is a global health challenge of increasing importance. The prevalence of multimorbidity (prevMM) is a central element in assessing the burden of disease in aging populations. Epidemiological studies of multimorbidity, most commonly defined as the co-occurrence of two or more chronic medical conditions (P2+), have been published for about 20 years now [1, 2]. Still, prevMM in most of these studies has only limited comparability due to the different study designs and definitions of multimorbidity used.

There are many aspects of the study design that can affect the comparability of prevalence estimates, such as the setting (general population, primary care, hospital, nursing home), the data source and collection (patient self-reports /interviews, medical reports, administrative data), the definition of prevMM, and the classification of diseases included. Any of these choices may influence prevalence estimates and thus affect comparisons between different populations at the same time (regional variations) or at different time points (trend estimates). In addition, demographic and socioeconomic factors are known determinants of multimorbidity [3, 4], especially age and gender [5, 6]. Nevertheless, estimates of prevMM vary widely across studies, and this impedes valid comparisons and the interpretation of differences between populations and subpopulations.

Reliable data on the prevalence of multimorbidity are urgently needed to inform medical and public health planning and for assessing the effects of medical and public health interventions. In recognition of these difficulties, the demand for a standardized operationalization of multimorbidity has been voiced recently [5, 7–10]. Among others, Fortin and colleagues have worked towards a more uniform definition and methodology [7, 11].

This study aims to make recommendations for a standard format in future studies for operationalizing predictors of prevMM. Our approach is empirical, as we analyzed data from our systematic review, relating P2+ simultaneously with the determinants mentioned above using meta analytic methods [12].

Our overall aim was to investigate which study design variables affect the measured prevalence of multimorbidity (P2+). These variables should be reported and possibly standardized in future studies. We therefore pursued two research objectives: (1) to identify a set of study design variables and demographic predictors of the prevalence of two or more chronic conditions, and (2) on the basis of (1), to make design recommendations for future studies of P2+ for optimal comparability.

## Methods

### Data collection

Data for this study were obtained through a systematic review of the literature. We screened for relevant articles published in English or German from January 1990 to December 2011 using PubMed/MEDLINE and Embase databases, CINAHL, the Web of Science and BIOSIS databases, and Google Scholar. For each database, search strategies with the terms multimorbidity or comorbidity or polymorbidity and variations of these (e.g., “multi-morbidity”) were used. We chose a lower boundary to focus on studies dealing with van de Akker’s concept of multimorbidity [13] and because publications on multimorbidity were rare before 2000 [14].

**Fig. 1:**
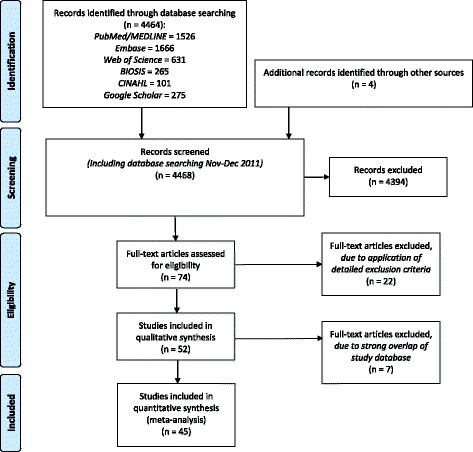
PRISMA flow diagram

The literature search was completed by screening the reference list of included articles. Details regarding the search strategy and the criteria defined for evaluation were described elsewhere [12]. Figure 1 shows the flow diagram of the evaluation process. We included only original studies addressing multimorbidity (two or more chronic medical conditions and no index disease or specific disease of interest) with a minimum sample size of 50 and – for homogeneity reasons – a study population with a majority of Caucasians.

The methods section of the studies included had to meet standards in research as in the STROBE statement for good reporting of observational studies [15]; in particular, the chronic conditions selected and the prevalence estimates had to be identifiable. Studies were also included when explicit prevalence estimates were missing but could be calculated from the information provided in the articles. The sample size of the study population as well as the setting had to be reported.

In this way, we compiled a database encompassing 52 studies. If the database of two studies strongly overlapped (in numbers and time frame), only the study deemed the more reliable was included in the analysis. For the present analysis, 45 studies allowing the estimation of P2+ remained. Key study characteristics are presented in Additional file 1. In total, 108 prevalence estimates were extracted from the 45 studies, one for each age group in each study. In these 108 age groups, gender was not assessed separately. Studies contributed between one and six age groups each.

Only seven studies presented prevalence estimates separately for men and women, resulting in 21 pairs (one for each sex) of age groups, (e.g., men aged 18–44, women aged 18–44). These data were used to investigate the gender effect. Age groups were the primary units of analysis. Mean age of an age group, if not available in the original article, was derived from age-specific population counts for the respective study year(s) in the Human Mortality Database [16].

### Classification of variables

Apart from the response variable P2+, the following variables were collected: origin/country of study population, setting (general population, primary care, hospital or nursing home, health insurance), data source (self-report, medical records, self-report combined with medical records, administrative data), length of data collection period, total sample size, number of age groups, range and mean age of each age group, number of individuals per age group, data reporting quality (data from original paper, calculated from numbers given in the paper, P2+ estimated from P3+ [12], or extracted from graph), and number of items in the classification of chronic conditions used.

We used two variables to characterize the disease classification: (1) a variable “disease classification,” characterizing the principle underlying the list of diseases considered, with (a) diseases from an internationally standardized disease classification coding system (e.g., ICD-10, ICPC-2) or (b) diseases described informally by the name of a disease (e.g., heart disease, diabetes), and (2) a variable “number of diseases in the classification,” counting the number of items in the classification used.

All 45 articles fulfilled at least 12 of the 22 possible quality criteria on the STROBE checklist (see Additional file 1) [15]. We applied the PRISMA checklist as far as possible (as described elsewhere [12]).

### Statistical analysis

Tables, percentages, medians, means and SD, and minima and maxima were used for descriptive analyses. Sign test as well as clustered regression (clusters = studies) were used for paired comparisons to investigate the gender effect. To assess the effects of the variables reported in Tables 1, 2 and 3, weighted regression models with a random effect at the level of age groups were used for logit P2+. These models account for unexplained variability between age groups, thus preventing spurious precision. We did several analyses, beginning with a detailed model similar to the model in Table 4 but with “number of diseases in the classification” with 22 levels and then successively combining variable categories with comparable effect to obtain a stable, not over-parametrized model. Models were assessed and variables were tested for significance using F-Tests; *p* < 0.05 was considered significant and *p* < 0.01 as highly significant. Graphs were used to visualize the effect of certain variables and to compare observed and fitted prevalence.

**Table 1:** Characteristics of studies used in the data analysis (*N* = 45)

Topic	Description	Number
Study characteristics	Studies contributing age groups	45
	Studies comprising several distinct substudies^a^	3
	Countries represented	17
Setting	General population	21
	Primary care practice	12
	Hospital/nursing home	3
	Health insurance	8
	Several settings (and different data sets)^b^	1
Disease classification	Name of diseases/disease groups only	30
	Diseases based on international classification systems	15
No. of diseases in the classification	Range of diseases under study	5 to over 300
	Median	16
Data collection period	Up to one year	25
	One year or more	19
	Several periods^c^	1
No. of study participants	Range of persons under study	301 to 5.6 million
	Median	6864
No. of age groups	1	26
	2 to 3	9
	4 to 6	10

^a^[19, 39, 40]

^b^[19]

^c^[39]

**Table 2:** Descriptive statistics for the age groups included

Items	N	Mean	SD	Min	Max
Limits					
Lower limit of age group given	104	50.7	24.5	0	85
Lower limit of age group imputed	4	15	n.a.	n.a.	n.a.
Upper limit of age group given	70	66.6	19.9	17	99
Upper limit of age group imputed	38	90	n.a.	n.a.	n.a.
Mean age^a^	108	60.3	20	9	89.3
Size					
No. of persons in age group	108	116,940	585,066	136	5,585,931
Prevalence					
P2+ (2 or more chronic conditions)	102	46.6%	24.4	0.3%	98.7%
P3+ (3 or more chronic conditions)	76	28.7%	22.0	0.0%	95.7%

^a^When mean age was not indicated, it was derived from the Human Mortality Database [16]

**Table 3:** Qualitative statistics for the age groups (*N* = 108)

Source of information	N	%
Source for P2+ (prevalence of 2 or more chronic conditions)		
Taken from paper	39	36.1%
Calculated from paper	55	50.9%
Extracted from graph	8	7.4%
Estimated using P3 + ^a^	6	5.6%
Source of age limits		
Both limits from paper	66	61.1%
Lower limit imputed	4	3.7%
Upper limit imputed	38	35.2%
Both limits imputed	0	0%

^a^Using Holzer et al.’s [12] method

**Table 4:** Variables and effect estimates from the model

Characteristics	Categories	Effect estimate	95% CI
Mean age	Years	0.052	0.044, 0.061
Number of age groups	1	0	–
	2	−2.7	−3.69, −1.71
	3	0.391	−0.14, 0.92
	4	0.474	−0.10, 1.05
	5	0.102	−0.47, 0.67
	6	0.001	−0.87, 0.87
Disease classification	Names of specific disease/disease groups	0	–
	Diseases based on ICD-10 or ICD-9 codes	1.26	0.039, 2.49
	Diseases based on ICPC-2 or CIRS	−0.789	−1.64, 0.067
No. of diseases in the classification	5–9	0	-
	10–24	0.516	0.0017, 1.03
	25–74	1.22	0.43, 2.01
	≥75	0.806	−0.47, 2.08
Setting	General population	0	–
	Primary care practice	0.015	−0.70, 0.73
	Hospital/nursing home	1.41	0.39, 2.44
	Health insurance	1.05	−0.66, 2.76
Data source	Self-report	0	–
	Medical record	−0.863	−1.71, −0.019
	Self-report + medical record	−0.75	−1.43, −0.071
	Administrative data	−0.497	−2.39, 1.39
Data collection period	Up to one year	0	–
	One year or more	−0.349	−0.79, 0.09
Data reporting quality	P2+ given in paper	0	–
	P2+ calculated from paper	−0.4	−0.94, 0.14
	P2+ read from graph in paper	−1.76	−2.52, −0.99
Constant		−3.305	−4.01, −2.60

Legend: Random effects model with response: logit P2+, sampling weights inverse to binomial variance of logit P2+; average sampling weight: 0.0182. *N* = 108; adjusted R2 = 70.6%; residual variance τ^2^ = 0.5812; overall F(20,87) = 12.61; *p* < 0.00005

Categories with “Effect estimate” = 0 are reference categories. Gender analyses were done using a different data set (see text). Mean age: Effect estimate gives the change in logit P2+ when changing mean age by one year. Other variables: Effect estimate quantifies the effect on logit P2+ of going from the reference category to the category of interest – e.g., going from one age group to two changes logit P2+ by −2.70, when keeping all other variables fixed (also see Discussion)

## Results

### Characteristics of the studies

Table 1 presents statistical information on the studies included in the data analysis. Our data set covered a wide variety of studies and included 17 countries and four different settings.

The number of single diseases listed ranged from five up to more than 300 items (single or grouped diseases), with a median number of 16 diseases. In each of the reviewed papers, an individual list of diseases/conditions was used for assessing multimorbidity (= two or more concurrent chronic conditions, P2+). In two out of three studies, the classification scheme was based on specific disease names/disease groups, whereas in the remaining studies the scheme was based on codes from an international classification of diseases system such as ICD-10 or ICPC-2. The number of participants per study ranged from 301 in the smallest study to 5.6 million in the largest. In 58% of the studies there was only one rather wide age group; other studies had up to six age groups.

### Descriptive statistics

Table 2 and 3 provide statistical information on the 108 age groups, the basic units of observation in this study. Salient points were: first, the large variability of the number of persons per age group, from 136 to 5.6 million (variation by a factor exceeding 40,000), and second, the wide variability of prevalence estimates found (e.g., from 0.3 to 98.7% for P2+).

Eighty-eight of 108 estimates of P2+ were either taken directly from the study paper or derived from numbers in the paper; six more were computed from separate estimates for men and women. Eight values of P2+ were extracted from graphs and another six calculated from the corresponding P3+ prevalence [12].

The gender effect was investigated by looking at the 21 pairs of P2 + -values available from seven studies presenting sex-specific figures for the same age group. Paired comparison showed that under similar circumstances (same age group, same study), women had a 3.0% higher prevalence P2+ than men (95% confidence interval [CI]: 0.84%, 5.2%, clustered regression). Women showed higher P2+ than men in 18 of the 21 prevalence pairs (*p* = 0.0015, sign test).

### Meta regression models

All other variables, including age, were investigated using meta regression models. A model incorporating all variables had a good fit (adjusted *R* ^2^ = 90.0%, residual unexplained variance = 0.197, F (45, 62) = 20.17, *p* < 0.00005) but showed clear signs of overfitting. Omitting insignificant variables and lumping together adjacent variables categories that showed almost identical effects, there resulted a model with 20 parameters that provided a reasonable fit (adjusted *R* ^2^ = 70.6%, residual variance = 0.5812, F(20, 87) = 12.61, *p* < 0.00005) and no evidence of overfitting. In this model, the following three determinants proved to be highly significant: “mean age” (t = 12.16, *p* < 0.0005), with a change in logit P2+ of 0.052 per year; “number of age groups” in the study (F(5, 87) = 9.82, *p* < 0.00005), and “data reporting quality” (F(2, 87) = 10.78, *p* = 0.0001). In addition, “disease classification” (F(2, 87) = 4.87, *p* = 0.01), “number of diseases in the classification” (F(3, 87) = 3.18, *p* = 0.03), and “setting” (F(3, 87) = 3.04, *p* = 0.03) proved to be significant determinants. On the other hand, the items “data collection period” and “data source” were not significant, with *p* values of 0.12 and 0.16, respectively. Table 4 presents the parameter estimates of this model.

**Fig. 2:**
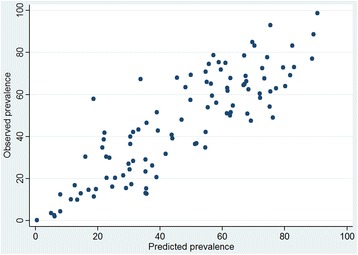
Observed versus predicted percentage of multimorbidity P2+ in a scatter plot

Figure 2 presents a scatter plot of observed and predicted prevalence estimates. Observed prevalence estimates cover the range from 0.3 to 98.7%, fitted estimates from 0.5 to 90.4%; the upper end was not fitted as well as the lower one. Differences ranged from −27 to 39%, with 50% between −10.5 and +8.8%, the mean deviation being 10.2%.

**Fig. 3:**
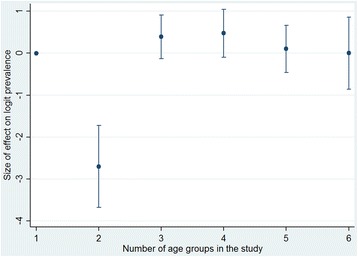
Effect of the number of age groups used in a study on multimorbidity P2+. Category “one age group” is the reference

Figure 3 shows the relative effect on logit P2+ of changing from a study with one age group to more than one age group. The maximum of P2+ is reached at four age groups. At two age groups, there is a dip in the effect. However, there were only two studies with two age groups.

**Fig. 4:**
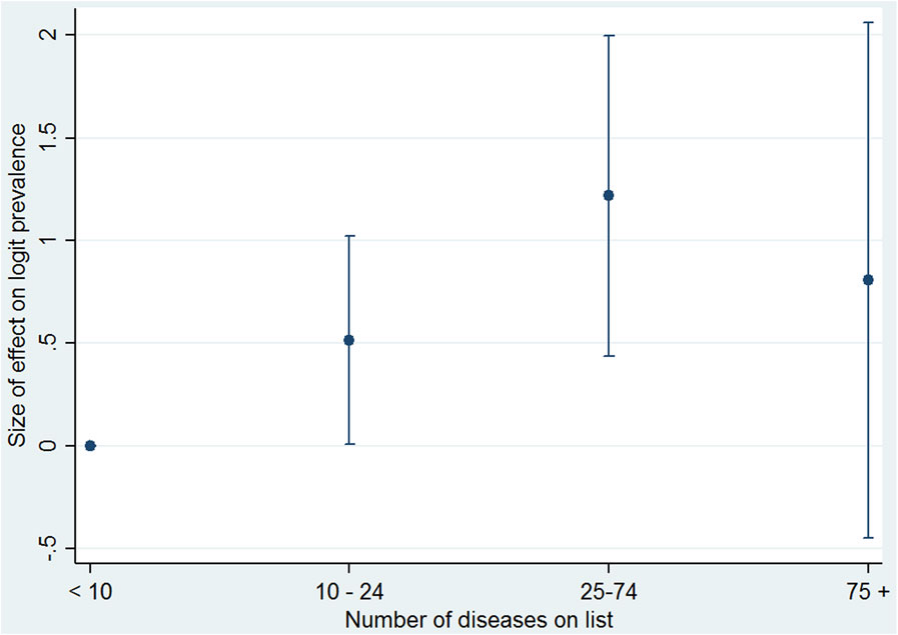
Effect of the number of diseases used in a study on multimorbidity P2+. Category “fewer than 10 diseases on list” is the reference

Figure 4 shows the relative effect on logit P2+ of going from a study with fewer than 10 diseases in the list used to studies with disease lists with higher numbers. The maximal prevalence P2+ is reached with lists of from 25 to 74 diseases, with a decrease with higher numbers of diseases in the classification.

Some of our findings may appear counter-intuitive at first sight, such as the finding that setting is not of high significance. This result states that with all other variables in the model, the additional contribution of the variable “setting” to an optimal fit is barely significant. Comparing the settings “primary care practice” and “hospital,” for example, our analysis does not state that there is no difference between those two settings in the level of P2+. However, adjusted for age, number of age groups in the study, data reporting quality, and disease classification used, the remaining differences in P2+ between the settings “primary care practice” and “hospital” almost disappeared. In the 45 studies available for analysis, the variables “data source” and “setting” were highly interdependent, e.g., health insurance databases contain administrative data only (data not shown).

## Discussion

Using data from a systematic review of 45 articles from 17 countries, we analyzed various variables regarding their impact on the prevalence estimates of multimorbidity, defined as the co-occurrence of two or more chronic conditions. To our knowledge this is the first study simultaneously considering population-related and design-related variables that influence P2+. As is well-known, age is an important determinant of the prevalence of multimorbidity [3, 5, 17]. So is gender, although that is less well-established [18]. We quantified the prevalence difference between men and women, with women showing on average a significant nearly 3% higher prevalence of P2+. This result agrees with women reporting higher prevalence of long lasting health problems (e.g., Swiss Statistical Office: https://www.bfs.admin.ch/bfs/de/home/statistiken/gesundheit/gesundheitszustand.html [18]). Less well known is the influence of study variables on the prevalence of multimorbidity such as the “number of age groups” (highly significant), “setting” (significant), and “number of diseases in the classification” used (significant) [5, 19, 20].

Before discussing these results, we would like to emphasize a few limitations in order to set the context of the validity of the findings of our study.

First, as in any systematic review, we cannot exclude bias in the search strategy used or resulting methodological bias due to the heterogeneity of the studies analyzed. A certain reassurance regarding these bias problems lies in the fact that our review is based on widely varying studies with differences in study design, instruments, scope, sample selection, assessed variables, and the language of the included studies, etc. Second, our basic units of observation were age groups, which entails several problems, such as the use of aggregated data. Thus, for example on the individual level, the age effect might be rather more pronounced than our result suggests. Third, we found considerable unexplained variation between studies, necessitating the use of random effect models. The correspondence of observed and fitted prevalences leaves room for considerable improvement. However, we believe that substantial improvement in fit can only be realized by improving the quality of studies of P2+. Thus, it might be useful to report study-specific regressions of prevalence of multimorbidity on age and gender in the future. Fourth, as in any multivariable analysis, interactions between the various variables are to be expected, which, with only 108 observations, we were unable to model. Therefore, our analysis could only provide a rough but we believe nevertheless useful description of the current situation regarding the influence of study design and demographic variables on prevMM. Finally, it has to be mentioned that, due to the scarcity of adequate data, we did not investigate the impact of socioeconomic status [4], the prevalence of disease patterns [21, 22], or the simultaneous use of two or more data sources [22, 23].

Despite these limitations, we believe that this paper contributes towards improving the design of multimorbidity studies.

Significant age and gender effects in multimorbidity have been reported in several studies [3–5, 17, 24–29]. In our study, age effect was substantial, too: average prevalence at mean ages 55 to 64 was 44.9%, whereas at mean ages 65 to 74, it was 51.3%, which is a relative increase of 14.2% within 10 years of age. Compared to this, the difference between women and men amounted to only 3.0% on average [18].

In our multivariable analyses, the “number of age groups included in the study” also had a highly significant effect on prevMM. The effect of number of age groups on P2+ was clear, but due to an outlier at two age groups, it is not easy to interpret. Here, further research may be needed. The implementation of age groups, in other words stratification by age, would allow better age adjustment and thus more precise comparisons of populations. To our knowledge, the use of age stratification was explicitly recommended by only one research group [30], although a priori epidemiological considerations would suggest it. Moreover, in our systematic review, 58% of all studies had only one, mostly wide age group. A few articles mentioned the impact of the study setting on prevMM [6, 19]. Differences in prevMM between the general population and primary care practices were described, the researchers stating that a health care setting can be expected to show higher prevalence than a general public setting. Our analysis showed that “setting” as such had only a marginal influence on prevMM – that is, differences in prevMM between settings such as those mentioned above were largely due to differences in age structure between the study populations and possibly some study design variables.

The item “data source” seemed to have no relevant effect in our analysis. In contrast, other studies found “data source” (e.g., self-reported data vs. administrative data) to have a significant influence on prevMM [19, 22, 31]. However, most of those studies did not suggest an adjustment for other important variables such as age and number of age groups.

In our analyses, “disease classification” and “number of diseases in the classification” had a significant effect on prevMM. Several research groups [5, 8, 32] have described the impact of the number of disease categories and pointed out the need for a consensus on a common classification of chronic conditions characterizing multimorbidity. Suggestions in this direction have been for a range of single diseases between 11 [33] and 30 [34]. Our results indicated that studies using classifications with fewer than 25 or more than 75 chronic conditions tended to yield lower prevalence estimates and thus confirmed a need to standardize disease classification to estimate prevMM. In our opinion, this choice should lead to the highest prevalence. Therefore, an upper limit is reasonable, because for more than 74 diseases, the effect of the number of diseases in the list on the fitted P2+ decreases again.

The “type of disease classification” – another study design variable not investigated in previous studies – had highly significant effects on prevMM in our analysis. This variable can be seen as a quality criterion to indicate whether the single disease entities were classified according to internationally accepted coding systems (e.g., ICD-10) or not. To quantify the burden of multimorbidity, it seemed sensible to us to suggest choices of design variables that maximize the resulting prevalence estimates. Therefore, we propose choosing a list of chronic conditions that contains from 25 to 75 single conditions.

Other authors have suggested classifications of similar size, such as the top 20 single diseases evaluated by Prados-Torres et al. [9] in their systematic review. The most frequent diseases were hypertension, COPD, diabetes, malignancy, stroke, dementia, depression, joint disease, anxiety, congestive heart failure, coronary heart disease, asthma, cardiac arrhythmia, thyroid disease, anemia, hearing problems, dyslipidemia, obesity, prostatic hypertrophy, and osteoporosis. In another systematic review, Sinnige et al. [35] assessed the top 20 diseases almost identically to Prados-Torres et al. In addition, Tonelli [34] identified a panel of 30 chronic conditions to be used in administrative data for which the best identified algorithm was of high or moderate validity. Alternatively, O’Halloran’s definition of chronicity could be useful as an underlying concept [36], as was applied by a Spanish and an Australian research group [32, 37].

To characterize multimorbidity, we suggest using the diseases identified in the studies named above. Such a core set of diseases and conditions could then be complemented by highly prevalent or critical chronic conditions relevant to the population under study.

The majority of the studies used in our analysis included the non-communicable chronic diseases that are highly prevalent in high income countries. But when looking at multimorbidity in a more global perspective or in low/middle income countries, other or additional relevant global chronic illnesses might have to be considered [38].

In recognition of some of the difficulties mentioned above, a need has been voiced lately for more uniform methods to enable solid comparisons between prevMM in different populations or over time [5, 7–10] and subsequently to create a solid database of prevMM. Data of that kind are urgently needed to inform medical and public health planning and, in the longer term, for assessing the effects of medical, public health, or other interventions. Criteria for a meaningful operationalization of multimorbidity, especially for epidemiological research, have been proposed by various researchers [7, 8, 19, 30]. Recently, an international research group advocated a method for validly identifying chronic conditions in administrative data [34] as a core set for designing observational studies.

According to our second research objective, based on our empirical evaluation and the considerations above, we derive recommendations regarding standards for future studies of the prevalence of multimorbidity. Thus, to enhance comparability of prevalence estimates, as well as to facilitate the combining of information from various studies (national and international), the following aspects should be considered in the planning of new epidemiological studies:

1. An overview of the population under study (gender, age range, setting, other socioeconomic variables) and the study design variables (total number of persons under study data source/data collection method, period of the data collection, etc.) should be standard for studies on the prevalence of multimorbidity.
2. Prevalence estimates should be given stratified by gender and age group to permit proper adjustment.
3. In the case of small databases or age-related limitations in the study design, stratification into at least three age groups with definite, pre-chosen upper and lower limits should be made. Alternatively, 10-year age groups could be considered, as practiced routinely by the World Health Organization.
4. The classification of chronic conditions used should comprise between 25 and 75 items. Both the name of the disease as well as the respective related code from an internationally accepted classification system should be documented.

## Conclusions

Our research revealed that at present, prevalence data on multimorbidity are less reliable than they could be. The main reasons for this are insufficient standardization and a lack of adequate control of key variables associated with the prevalence of multimorbidity. Our suggestions for increasing the comparability of prevalence data in future studies were guided by a pragmatic empirical approach and aimed at facilitating the implementation of a uniform methodology. We expect that we can contribute to progress towards a more uniform definition of multimorbidity and its prevalence. Reliable and thus comparable data of multimorbid populations are urgently needed, not only in order to identify the magnitude of the problem but also to measure intervention effects in populations. To achieve this goal, a consensus on the operationalization of multimorbidity and prevalence of multimorbidity has to be reached. This paper provides an empirical basis for that consensus.

## Additional file

Additional file 1: Studies included in the analyses (*N* = 45) with complete reference information for the studies listed in the table. (PDF 62 kb)

## Abbreviations

P2+: Prevalence of two or more concurrent chronic medical conditions
P3+: Prevalence of three or more concurrent chronic medical conditions
prevMM: Prevalence of multimorbidity (generic term)
STROBE: Strengthening the Reporting of Observational Studies in Epidemiology
