# Supplementary material for: Evidence-based design recommendations for prevalence studies on multimorbidity: improving comparability of estimates
Source: Popul Health Metr. 2017 Mar 7;15:9. doi: 10.1186/s12963-017-0126-4 (PMC5341353; doi:10.1186/s12963-017-0126-4)
Supplement: Additional file 1: — Studies included in the analyses (N = 45) with complete reference information for the studies listed in the table. (PDF 62 kb) [file 12963_2017_126_MOESM1_ESM.pdf]

Additional file 1. Studies included in the analyses (N=45) with complete reference information for studies listed in the table.

No. of study	Name (Year)	STROBE Criteria* (total score)	Study Population	Setting	Data source	Data collection period	No. of patients in study	Age group	Mean age	No. of patients per agegroup	No. of diseases in the classif.	Disease classifi-cation
1	Alonso et al 2004	21	General Social Surveys, United States, 1989-90	General population	Self report	Period prevalence	2474	≥18	43.6	2474	11	Morbidities
			Central Person Registry data, Denmark, 1994	General population	Self report	Point prevalence	4084	≥16	43.9	4084		
			Sofres METASCOPE database, France, 1995	General population	Self report	Point prevalence	3656	≥15	44.6	3656		
			Infratest National database, Germany, 1994	General population	Self report	Point prevalence	2914	≥14	45.2	2914		
			National sample lists, Italy, 1995	General population	Self report	Point prevalence	2031	≥18	46.0	2031		
			National and Amsterdam registry, The Netherlands, 1994 & 1996	General population	Self report	Period prevalence	4059	≥12	43.4	4059		
			Office of the National Registrar, Norway, 1996	General population	Self report	Point prevalence	2323	19-80	44.9	2323		
2	Aarts et al 2011	21	Postal survey Limburg, The Netherlands, 2008	General population	Self report	Point prevalence	15188	55-90	67.7	15188	22	Morbidities
3	Britt et al 2008	18	General Practice Substudy of Beach program, Australia, 2005	Primary care practice	Self report & medical record	Point prevalence	9111	<25	12.8	2047	17	Morbidities
								25-44	35.2	2281		
								45-64	54.1	2450		
								65-74	69.7	990		
								≥75	81.8	1343		
4	Caughey et al 2010	19	Longitudinal study of ageing, Australia, 1992 - 06	General population	Self report	Period prevalence	2088	65-69	67.4	136	17	Morbidities
								70-74	72.4	558		
								75-79	77.4	522		
								80-84	82.3	436		
								>85	89.0	435		
5	Cesari et al 2006	21	Population based ilSIRENTE study, Italy, 2003	General population	Self report & medical record	Point prevalence	364	≥80	85.9	364	14	Morbidities
6	Chan et al 2002	14	Hospital admissions Randwick Area, Australia, 1998-99	Hospital/nursing home	Self report & medical record	Period prevalence	526	55-95	68.5	526	10	Morbidities
7	Fortin et al 2005	20	Patients recruited from Quebec, Canada, 2003	Primary care practice	Medical record	Point prevalence	980	18-44	32.2	231	~300	ICPC-2
								45-64	53.7	429		
								≥65	75.3	320		
8	Fortin et al 2010	20	Canadian Health Survey, Canada, 2005	General population	Self report	Point prevalence	26000	25-44	35.6	11186	7	Morbidities
								45-64	53.9	9870		
								65-79	71.9	3647		
								80+	85.3	1296		
9	Fuchs et al 1998	21	National Population Register (NPR) and CALAS Study, Israel, 1998-92	General population	Self report	Period prevalence	1487	75-94	80.9	1487	14	Morbidities
10	Fung et al 2008	19	Telephone survey CQI Study, United States, 2001/02	General population	Self report	Period prevalence	15709	18-97	45.8	15709	16	Morbidities
11	Galenkamp et al 2011	21	LASA population-based Study, The Netherlands, 2005/06	General population	Self report	Period prevalence	2046	57-98	69.2	2046	9	Morbidities
12	Gunn et al 2010	20	Diamond Longitudinal Study, various GPs, Australia, 2005	Primary care practice	Self report	Point prevalence	6864	18-44	31.8	2453	12	Morbidities
								45-64	54.1	3142		
								≥65	75.4	1200		
13	Glynn et al 2011	20	3 primary care practices West Ireland, Ireland, 2008/09	Primary care practice	Medical record	Period prevalence	3309	50-59	54.8	1309	147	ICPC-2
								60-69	64.5	975		
								70-79	74.6	628		
								≥80	85.4	397		

14	Hoffman et al 1996	18	National Medical Expenditure Survey, United States, 1987	General population	Self report	Point prevalence	27505	0-17	9.0	7178	~1000	ICD-9
								18-44	30.9	11889		
								45-64	54.8	5103		
								≥65	74.5	3335		
15	Hudon 2008	20	Quebec Health Survey, Netherlands, 1998, Canada	General population	Self report	Point prevalence	16782	18-69	41.2	16782	25	Morbidities
16	Hung et al 2011	20	Health and Retirement Study, United States, 2008	General population	Self report	Point prevalence	11321	65-69	67.4	3128	7	Morbidities
								70-74	72.4	2908		
								75-79	77.4	2180		
								80-84	82.4	1517		
								≥85	89.2	1588		
17	Jansa et al 2010	20	Hospital inpatients Barcelona, Spain, 2004	Hospital/nursing home	Self report & medical record	Point prevalence	301	23-93	49.1	301	40	ICD-9
18	Laux et al 2008	16	24 GPs in Germany using Content Database, Germany, 2006	Primary care practice	Medical record	Point prevalence	39699	≤50	28.5	18815	147	ICPC-2
19	Lee et al 2008	17	Veteran Health care, United States, 1999-00	Health insurance	Administrative data	Period prevalence	741847	55-64	59.6	741847	11	ICD-9
20	Loza et al 2009	20	EPISER Health survey, Spain, 1999/00	General population	Self report	Period prevalence	2192	≥20	47.5	2192	9	Morbidities
21	Macleod et al 2004	13	Cairns practice registry, United Kingdom, 2000	Primary care practice	Medical record	Point prevalence	7286	≥18	47.7	7286	17	Morbidities
22	Marengoni et al 2008	14	Population based Kungsholmen Project, Sweden, 1987- 2000	General population	Medical record	Period prevalence	1099	77-84	80.6	306	30	ICD-9
								≥85	88.8	296		
23	Menotti et al 2001	15	FINE Study, Finland, 1984-94	General population	Self report & medical record	Period prevalence	2285	65-84	72.6	716	7	Morbidities
			FINE Study, The Netherlands, 1985-95	General population	Self report & medical record	Period prevalence		65-84	72.6	887		
			FINE Study, Italy, 1985-95	General population	Self report & medical record	Period prevalence		65-84	72.8	682		
24	Min et al 2007	19	ACOVE Study (Managed Care), United States, 1998-99	Health insurance	Medical record	Period prevalence	372	≥65	75.3	372	8	Morbidities
25	Minas et al 2010	19	Various primary health care centers, Central Greece, 2008	Primary care practice	Self report & medical record	Point prevalence	20299	14-64	50.0	8423	~300	ICPC-2
26	Murtaugh et al 2009	16	OASIS (MediCare, MediCaid), United States, 2004 & 2005	Health insurance	Administrative data	Period prevalence	5585931	≥65	75.6	5585931	18	ICD-9
27	Naessens et al 2011	14	Various administrative data, United States, 2004 - 07	Health insurance	Administrative data	Period prevalence	33324	18-34	26.4	9170	56	ICD-9
								35-49	42.6	14607		
								50-64	56.7	9547		
28	Nagel et al 2008	20	EPIC-Heidelberg cohort, Germany, 1994-98	General population	Self report & medical record	Period prevalence		50-74	61.4	13781	15	Morbidities
29	Nägga et al 2012	20	Health survey Linköping, Sweden, 2007/08	General population	Self report & medical record	Period prevalence	496	85	85.9	496	14	ICD-10
30	Naughton et al 2006	17	Pharmacy Database, Ireland, 2004	Health insurance	Administrative data	Point prevalence	316928	≥70	78.2	316928	9	Morbidities
31	Newacheck et al 1991	16	National health interview survey on child health, United States, 1988	General population	Self report	Point prevalence	7465	10-17	14.1	7465	19	Morbidities
32	Rapoport et al 2004	13	National Population Health Survey, Canada, 1998-99	General population	Self report	Period prevalence	13682	20-39	30.6	5308	24	Morbidities
								40-59	48.8	4720		
								60-79	69.0	2995		
								≥80	85.3	659		
33	Salisbury et al 2011	18	UK General Practice Research Database, United Kingdom, 2005 - 08	Primary care practice	Medical record	Period prevalence	99997	≥18	48.0	99997	17	Morbidities
34	Schellevis et al 1993	16	Different GP practices, The Netherlands, 1988	Primary care practice	Medical record	Point prevalence	23534	<65	30.8	21349	5	Morbidities
35	Schneider et al 2009	17	CCW MediCare, United States, 2005	Health insurance	Administrative data	Point prevalence	1649574	≥0	36.9	1649574	6	Morbidities
36	Schram et al 2008	17	Leiden 85-plus population-based Study, The Netherlands, 1997	General population	Self report & medical record	Point prevalence	599	≥85	88.9	599	12	Morbidities
			Rotterdam population-based Study, The Netherlands, 2002	General population	Self report & medical record	Point prevalence	3550	65-99	75.0	3550	14	Morbidities
			January 1, 2005 10 years of registration time CMR, GP registries in Nijmegen, NL	Primary care practice	Self report & medical record	Point prevalence	2895	≥55	68.0	2895	72	Morbidities
			RNUGP, GP registries Leiden region, The Netherlands, 2006	Primary care practice	Self report & medical record	Point prevalence	5610	≥55	68.0	5610	83	Morbidities
			LMR Hospital register, The Netherlands, 2003-04	Hospital/nursing home	Medical record	Point prevalence	1058234	≥55	68.0	1058234	185	ICD-9
			RAI Nursing home, The Netherlands, 2005	Hospital/nursing home	Medical record	Point prevalence	1274	≥55	68.0	1274	26	Morbidities

37	Taylor et al 2010	19	North West Adelaide Health Study, South Australia, 2004- 06	General population	Self report & medical record	Period prevalence	3203	20-39	30.1	1226	7	Morbidities
								40-59	49.5	1171		
								60-90	72.0	808		
38	Tucker-Seeley et al 2011	21	Population based Health and Retirement Study, United States, 2004	General population	Self report	Point prevalence	7305	50-59	54.7	2004	6	Morbidities
								60-69	64.6	3218		
								≥70	78.8	2083		
39	Uijen et al 2008	15	CMR Nijmegen, The Netherlands, 2005	Primary care practice	Medical record	Point prevalence	13584	45-64	54.3	8764	~300	ICPC-2
								65-74	69.7	3067		
								>75	81.7	1753		
40	v.d.Akker et al 1998	17	Registration Network Family Practices (RNH), The Netherlands, 1994	Primary care practice	Medical record	Point prevalence	60857	<20	9.9	13717	~300	ICPC-2
								20-39	30.0	19121		
								40-59	49.0	16064		
								60-79	68.8	10335		
								≥80	85.1	1620		
41	v.d.Bussche et al 2011	21	GEK claims data, Germany, 2004	Health insurance	Administrative data	Point prevalence	123224	≥65	74.7	123224	46	ICD-10
42	Walker et al 2007	17	National Health Survey 2001, Australia, 2001	General population	Self report	Point prevalence	17450	20-39	30.3	6664	8	Morbidities
								40-59	49.2	6427		
								60-74	67.0	2887		
								>75	81.5	1472		
43	Wang et al 2008	21	Different GP practices, Germany, 2002	Primary care practice	Self report & medical record	Point prevalence	1009	15-89	46.9	1009	13	Morbidities
44	Wolff et al 2002	19	Medicare Part A and B, United States, 1999	Health insurance	Administrative data	Point prevalence	1217103	65-69	67.5	442583	23	MDC**
45	Wong et al 2011	21	Dutch Hospital Register (LMR) 2004, The Netherlands, 1995 - 04	Hospital/nursing home	Medical record	Period prevalence	1414142	≥0	38.1	1414142	138	Morbidities

* Based on Strengthening the Reporting of Observational studies in Epidemiology (STROBE), checklist for reporting of observational studies

** MDC: Major Diagnostic Category; ICD-9, ICD-10: International Classification of Diseases; ICPC-2: International Classification of Primary Care
